# Health of single mothers and fathers in Germany. Results of the GEDA studies 2019 – 2023

**DOI:** 10.25646/12193

**Published:** 2024-07-03

**Authors:** Petra Rattay, Yasmin Öztürk, Raimund Geene, Stefanie Sperlich, Ronny Kuhnert, Hannelore Neuhauser, Ulfert Hapke, Anne Starker, Claudia Hövener

**Affiliations:** 1 Robert Koch Institute, Department of Epidemiology and Health Monitoring, Berlin, Germany; 2 Heinrich Heine University Düsseldorf, University Hospital, Institute for Medical Sociology, Düsseldorf, Germany; 3 German Youth Institute, Munich, Germany; 4 Alice Salomon University of Applied Sciences, Berlin, Germany; 5 Berlin School of Public Health, Berlin, Germany; 6 Hannover Medical School, Medical Sociology, Hannover, Germany

**Keywords:** Single parents, Single-parent family, Family type, Health, Health inequalities

## Abstract

**Background:**

The living situation of single parents is often characterised by sole responsibility for family and household, problems in reconciling work and family life, and a high risk of poverty. In a comparative perspective with parents in partner households, the health of single mothers and fathers was analysed, considering differences in their social status.

**Methods:**

The analyses are based on data from the GEDA studies 2019 – 2023 (7,999 women, 6,402 men). Prevalences for single mothers and fathers and mothers and fathers living in partner households were calculated for self-rated health, chronic diseases, depressive symptoms, smoking and utilisation of professional help for mental health problems. In multivariate models, adjustments were made for income, education, employment status and social support, and interactions with family type were included.

**Results:**

Single mothers and fathers show higher prevalences for all health indicators in comparison to parents living in partner households. Also after adjustment, the differences between family types remain significant. The health of single mothers also varies partially with income, employment status and social support.

**Conclusions:**

Health promotion measures have to consider that single parents are a heterogeneous group. In addition to strengthening personal skills, policy and setting-based interventions aim to reduce health inequalities.

## 1. Introduction

The Federal Government’s Tenth family report ‘Support for single and separated parents and their children – stocktaking and recommendations for action’ covers many aspects of the living situation of separated families [3]. It also looks at the health situation of single and separated parents. This article describes various aspects of the health of single parents (Infobox) for the Tenth family report, based on current cross-sectional data from the German Health Update (GEDA) for the years 2019 to 2023.

In 2022, 1.33 million single mothers and 239,000 single fathers in Germany lived together in a household with at least one minor child [4], which constitutes in 18.5 % of all families. In total, 2.26 million children under the age of 18 lived with a single parent, with the majority of children living in the mother’s household (85.6 %) [4].

Today, single-parent families represent a socially established family form alongside others, where family forms among single parents and the care models of separated families are becoming increasingly differentiated and pluralised [2, 5]. However, in a survey conducted in 2020, only 12 % of single parents reported a balanced division of responsibility for their children between the separated parents [5].

**Infobox About the term ‘single parent’**

The German Federal Statistical Office defines ‘single parents’ as ‘mothers or fathers who live in a household with minor or adult children without a spouse or partner’ [1], although official statistics often restrict the definition to households with at least one minor child. In social law, single parents are defined as ‘persons who live together with one or more minor children and are solely responsible for their care and upbringing’ (§ 21 (3) SGB II). The term is also widely used in research – albeit with different operationalisations. However, lately the term ‘single parent’ has come to be viewed critically, as many mothers and fathers share the upbringing and care of their children in different ways even after separation. As a result, the term ‘separated parents’ has also been introduced. As the GEDA studies do not differentiate between single and separated parents, this article uses the term ‘single parents’ as a generic term for mothers or fathers living together in a household with at least one minor child, but without a partner [1], knowing that the term does not adequately reflect the diversity of the different family types among separated and single parents [2].

Single parents are characterised by a great heterogeneity in their living situations. They differ not only in terms of their family situation, the care arrangements made with the other parent and the reasons for single parenting (separation from or death of the other parent or deliberate single parenting), but also in terms of sociodemographic characteristics, socioeconomic situation, personal, social and time resources as well as the duration of single parenting [2, 5].

Due to the predominantly sole responsibility of one parent for child rearing and care, household and employment [5] the social situation of the majority of single parents differs in some respects from parents in partner households. Single parents often find it much more challenging than parents from partner households to reconcile their family and work lives [2, 5]. In order to generate a sufficient household income, single mothers are more likely to engage in full-time employment than mothers living in partner households. Conversely, single fathers are more likely to be in part-time employment or not in work at all due to their family responsibilities in comparison to fathers in partner households [5, 6]. In addition, the living situation of single parents is often characterised by financial strain [2, 4]. In 2021, 41.6 % of single-parent households had a monthly income that fell below the at-risk-of-poverty threshold [7]. A total of 34.6 % of single-parent households received benefits under the German Social Code Book II (SGB II) in 2021, while this only applied to 6.6 % of two-parent households [7]. Among single parents with three or more children, the proportion of those receiving benefits under SGB II was 86.2 % [7].

During the COVID-19 pandemic, these pre-existing structural challenges faced by single parents were further exacerbated [2, 5]. The various containment measures, including contact restrictions, childcare facility closures, homeschooling, and remote work arrangements, have placed single parents in a challenging position. This is particularly evident in instances where they are required to balance work and family responsibilities, such as household chores and childcare, or when they experience income losses due to unemployment or reduced work opportunities [8]. Accordingly, studies have found that single mothers have experienced a significantly higher overall burden during the course of the COVID-19 pandemic than mothers in partnerships [2, 5, 8–10].

In general, single parents are often confronted with particular challenges due to limited social, temporal, and financial resources [5, 11] which can be reflected in their health status.

A multitude of studies conducted in Germany have demonstrated that the mental health of single mothers, in particular, [12–19] and fathers [12, 14, 19] is more frequently impaired than that of parents living in partner households. In the context of the pandemic, only a limited number of studies have examined the mental health of single parents in comparison to parents living in a partnership. A higher level of psychological distress was observed in single parents during the period from March 2020 to April 2021 [20]. Furthermore, an increase in exhaustion was noted in the spring of 2020 in comparison to the period prior to the pandemic [21]. The elevated stress levels and increased loneliness among single parents, which were previously documented prior to the pandemic, persisted throughout the pandemic [21]. With regard to anxiety, no significant differences were observed between single parents and parents in partner households at the beginning of the pandemic [22].

Furthermore, studies have indicated that single mothers [18, 23–25] reported poorer general health than mothers in a partnership. Among fathers, the differences by family type are not statistically significant [14]. The AOK Family Study 2022 has demonstrated that single parents experience poorer general health than parents from partner households during the pandemic [26].

In terms of physical health, however, differences between family types were found for the pre-pandemic period only in general physical complaints [24, 27] as well as in specific health indicators such as chronic back pain [14, 18] or joint pain [18].

In addition, single mothers [14, 25, 28, 29] and fathers [14] are more likely to smoke than parents in a partnership. Furthermore, single mothers are also more likely to engage in risky alcohol consumption [30] and are less active in sports [14] whereas for fathers, the differences by family type are not significant. Single parents also report more frequently utilisation of counseling or other support services for families [31] as well as psychotherapy [32]. However, there are no discernible differences in the utilisation of other specialist medical practitioners according to family type [32]. There are no studies on the health behavior of single parents in Germany during the pandemic.

Burdens resulting from socioeconomic disadvantages and lack of social support are discussed as possible explanations for the poorer health and lower levels of health-promoting behaviour observed in single parents [18, 33, 34]. However, studies for Germany show that greater health impairments among single parents can be attributed in part, but not entirely, to differences in socioeconomic situation and social support [13, 14, 19, 25]. Studies also show that single parents are not a homogeneous group, but that there are sometimes large differences in health depending on the financial situation, educational attainment, employment status, or social support [14, 18, 35–37].

Although the COVID-19 pandemic has often led to major financial and psychosocial burdens, especially for single parents [2, 5, 8–10], only limited research has been carried out in Germany on the health situation of single parents under the societal conditions of recent years [20–22, 26]. The available studies mainly relate to mental health and to limited periods of time during the pandemic, are based on small numbers of single parents and are characterised by a lack of representativeness.

This article therefore aims to analyse the current health situation of single parents (a) based on different health outcomes with high public health relevance in the age group of 18 to 59 years (b) for both single mothers and single fathers and (c) considering the diversity of social living conditions of single parents and parents living in partner households.

The article addresses the following questions in detail:

1. Are there differences between single mothers/fathers and mothers/fathers living in partner households in terms of social situation, health and health behaviour?
2. Can the differences in health and health behaviour between single mothers/fathers and mothers/fathers living in partner households be explained by differences in income, education, employment status or social support?
3. Do the associations between the family type (single-parent household vs. partner household) and the health or health behaviour of mothers vary with income, employment status and social support?

## 2. Methods

### 2.1 Data

The German Health Update (GEDA) is a nationwide cross-sectional survey of the resident population aged 15 and over living in Germany and has been carried out regularly by the Robert Koch Institute on behalf of the Federal Ministry of Health since 2008. For the present analysis, the data from the GEDA 2019/2020, GEDA 2021 and GEDA 2022/2023 studies were pooled (versions from February 8, 2024). Overall, data is available for the period from April 2019 to November 2023 (except for a break from January to June 2021).

The GEDA study is conducted as a telephone survey using a programmed, fully structured questionnaire (Computer Assisted Telephone Interview, CATI). Participants are selected using a random sample of landline and mobile phone numbers (dual-frame method) [38]. The population comprises the population aged 15 and over living in private households whose usual place of residence is in Germany at the time of data collection. The sample sizes and response rates for the individual GEDA waves can be found in Annex Table 1. A total of 90,671 people participated in the included GEDA waves. A detailed description of the methodology of the GEDA study can be found in Allen et al. [39].

Only people living in households with at least one own child under the age of 18 were included in the present analysis. No differentiation was made between biological children, stepchildren and adopted children (social parenthood). Individuals below the age of 18 and above the age of 60 were excluded, as the proportion of individuals with underage children in these age groups is low. After plausibility checks of the age information of the household members, 50 respondents were excluded. Consequently, data is available for the age group 18 to 59 years from 7,999 mothers and 6,402 fathers. Of these, 1,276 are single mothers and 339 are single fathers.

### 2.2 Variables

#### Outcome variables

The self-rated general health status was collected using the question ‘What is your general state of health like?’ The five response categories were grouped into very good/good (good) and fair/poor/very poor (not good). The prevalence of a health status assessed as not good is reported below.

With regard to the presence of a chronic illness (yes/no), participants were asked the following question in the questionnaire: ‘Do you have a chronic disease or a long-term health problem? This refers to diseases or health problems that have lasted or are expected to last for at least 6 months.’

The presence of depressive symptoms (yes/no) within the last two weeks was recorded using the Patient Health Questionnaire-2 (PHQ-2) as a self-report by the respondents. The question was: ‘Over the last 2 weeks, how often have you been bothered by the following problems?’ The PHQ-2 comprises the first two items of the PHQ-9: ‘Little interest or pleasure in doing things’ and ‘Feeling down, depressed or hopeless’. The response categories are: not at all (0), several days (1), more than half the days (2), nearly every day (3). For the PHQ-2 score the values of both items were added together. The PHQ-2 total score ranges therefore from 0 to 6. If the score is 3 or greater, depressive symptoms are likely [40].

Information on smoking (yes/no) was collected using the question ‘Do you smoke tobacco products, including tobacco heaters? Please exclude electronic cigarettes or similar products.’ For the purpose of this analysis, the response categories ‘daily’ and ‘occasionally’ (yes) as well as ‘no, not anymore’ and ‘I have never smoked’ (no) were combined.

With regard to the utilisation of professional help due to psychological distress, the self-assessed need for professional help (yes/no) was considered. Participants were asked the following question: ‘In the previous 12 months, have you ever had the impression that you should seek professional help due to problems with your feelings, nervous distress or alcohol or drug use?’ Additionally, the self-reported use of professional help (yes/no) was recorded using the question ‘In the previous 12 months, have you sought professional help due to problems with your feelings or nervous distress or alcohol or drug use?’ The two variables were only surveyed from February 2022 onwards and only in a sub-sample.

#### Predictor variable

In order to identify single mothers and fathers, the partner status is used, which indicates whether a partner resides in the same household as the respondent (single-parent household vs. partner household). Here, it is irrelevant whether other individuals (such as adult children, the respondent’s parents, etc.) residing in the household in addition to the partner and underaged children. The marital status and gender of the partner are not considered [cf. 6].

#### Stratification, control and mediator variables

The gender of the respondents (female/male) was included in the analysis as a stratification characteristic. Gender identity was used for this purpose, as measured by the question ‘Which gender do you feel you belong to?’ Due to the small number of cases, information on non-binary individuals is not shown.

Adjustments were made for the control variables age (18 – 29 years, 30 – 39 years, 40 – 49 years and 50 – 59 years), region of residence (West Germany, East Germany and Berlin), country of birth (Germany vs. not Germany) and year of survey. In addition, the number of minor children in the household (one child, two children and three or more children) and the age of the youngest child in the household (0 – 6 years, 7 – 10 years and 11 – 17 years) were included, as these vary greatly between the family forms [4].

The mediator variables considered were income, educational level, employment status and social support. For income, household equivalised income was used to account for differences in household composition. This was divided into low (< 60 %), medium (60 % – < 150 %) and high (≥ 150 %) using the median income in 2021 [41]. With regard to the respondents’ level of education, the Comparative Analysis of Social Mobility in Industrial Nations (CASMIN) classification was used to classify the highest formal education level into low, medium and high [42]. In terms of employment status, a distinction was made between full-time, part-time and not employed (including unemployed, students, pensioners and persons doing voluntary work or housework). The classification was made by the respondents themselves. Social support was measured using the Oslo 3 Item Social Support Scale [43] which allows categorisation into low, medium and strong support.

### 2.3 Statistical analysis

The first step was to calculate stratified prevalences by gender for the social determinants and each health outcome for single mothers and fathers and those living in partner households (research question 1). In addition, the p-value of the Chi^2^ test is reported.

In the second step, prevalence ratios (PR) were determined for all health outcomes using Poisson regressions including all control variables (model 1) and the stepwise inclusion of the mediator variables. Finally, the PRs for the fully adjusted models are reported. In this way, it can be seen whether the differences in health and health behaviour between single mothers and fathers and those living in partner households found in the descriptive analysis remain significant even after controlling for sociodemographic and social determinants (research question 2).

In the third step, predictive margins and predicted probabilities were calculated for the mothers using Poisson regressions (model 1: adjusted for the control variables) with interaction terms from (a) partner status and income, (b) partner status and employment status, and (c) partner status and social support. The predicted probabilities, which can be interpreted as adjusted prevalences, are presented graphically (research question 3). The outcome variables included in this analysis step were self-rated health, depressive symptoms, smoking and the utilisation of professional help due to mental health problems.

Only cases with complete information on control and mediator variables were included in the multivariate modelling.

P-values lower than 0.05 were considered as statistically significant. In addition, the 95 % confidence intervals are reported.

All calculations were performed with a weighting factor that corrects for deviations of the sample from the population structure with regard to age, sex, level of education and federal state [39]. Due to the modular survey design in GEDA 2022/2023, two weighting factors were calculated for these waves. One weighting factor is for variables that were collected in the core survey and thus in each module, and a second weighting factor is determined for calculations with variables from individual modules that were not continuously surveyed in GEDA 2022/2023. The level of detail of the adjustment weights differs between the GEDA survey waves due to the different sample sizes. For GEDA 2022/2023, only a rough adjustment to the population can be made. Age groups and federal states have been combined to achieve a minimum number of respondents per weighting cell. The analyses were performed with the statistical software StataSE 17 (StataCorp, College Station, TX, USA). The description of the sample can be found in Annex Table 2.

## 3. Results

In the GEDA studies for the years 2019 to 2023, 18.0 % of mothers and 5.5 % of fathers reported being single parents. The social and economic situation of single mothers and fathers differs significantly from that of mothers and fathers living in partner households (research question 1, Table 1). Both single mothers and single fathers are more likely to have only one minor child living in the household, and the youngest child of single parents is older than that of parents living with a partner. Single mothers and fathers have a lower monthly household income at their disposal and feel less socially supported than mothers and fathers living in partner households. Single mothers are also more likely to have a low level of education and are more likely to be in full-time employment than mothers in partner households, while single fathers are less likely to be in full-time employment than fathers in partner households. For fathers, there are no differences in education between family types.

For single mothers and single fathers, the prevalence of all health indicators is significantly higher than for mothers and fathers living in a partner household (research question 1, Table 2).

In the Poisson regressions, for all health outcomes, also after controlling for age, region of residence, country of birth, year of survey, number of children and age of the youngest child (model 1), significantly higher prevalence ratios (PRs) are found for single mothers (Table 3) and single fathers (Table 4) compared to mothers and fathers living in a partner household.

In the following models, a mediator variable was included in addition to the control variables in order to test whether the differences found in model 1 persist when adjusting for the mediator variables (research question 2).

In the case of self-rated health, chronic diseases and depressive symptoms, the largest decline in PRs can be seen among mothers when adjusting for income. However, the adjustment for level of education and social support is also associated with a reduction in PRs for mothers. For fathers, the strongest decline in PRs was recorded for these three outcomes when social support and employment status were included, followed by income.

For smoking, including level of education and income leads to a decline in PRs for single parents for both mothers and fathers.

Regarding the need for and utilisation of professional help, there is a decrease in the PRs for mothers when adjusting for social support and for fathers when adjusting for social support and employment status.

However, even after adjusting for all control and mediator variables, single mothers and fathers have significantly higher PRs for all outcomes except chronic disease.

The differences reflected in the unadjusted prevalences can thus be attributed in part, but not entirely, to the different social composition of the groups of single mothers and fathers and of mothers and fathers living in partner households. Only in the case of chronic diseases can the differences found by family type be fully explained by the social determinants.

**Figure 1:**
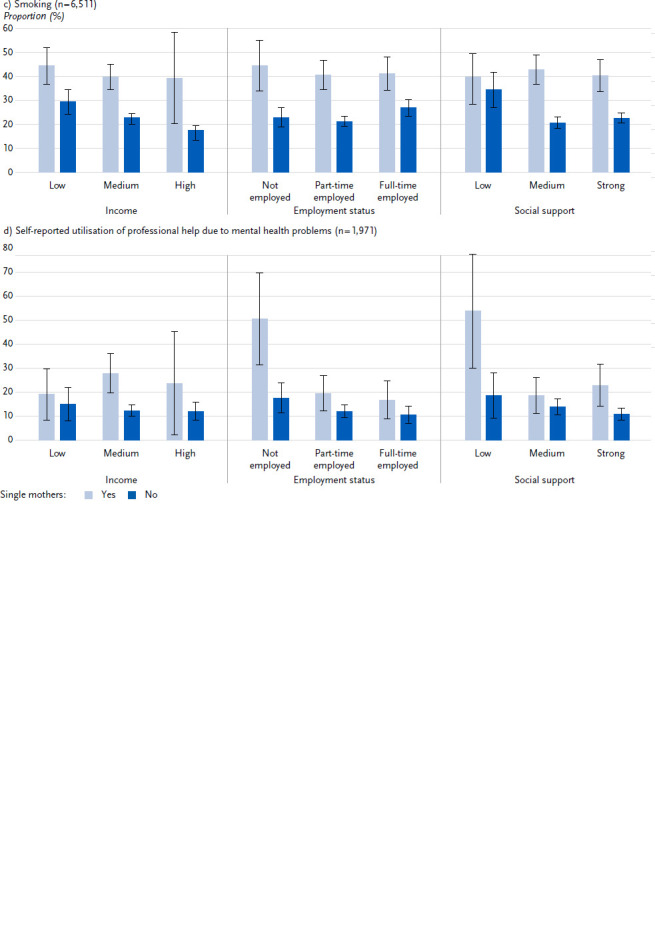
Health and health behaviour of single mothers compared to mothers living in partner households, stratified by income, employment status and social support (predicted probabilities in %, 95 % confidence intervals, Poisson regressions with interactions on partner status, all models adjusted for age, region of residence, country of birth, year of survey, number of children and age of youngest child). Source: GEDA 2019/2020, GEDA 2021, GEDA 2022/2023 (pooled)

With regard to research question 3 of whether the associations between family form and health status vary with income, employment status or social support (Figure 1), the income gradient for self-rated health and depressive symptoms is more pronounced among single mothers than among those living in partner households. The highest adjusted prevalences for poor-rated general health and depressive symptoms are found among single mothers in the low-income group. Mothers in the high-income group are less likely to report poor general health or depressive symptoms than mothers in the low-income group, irrespective of partner status.

In terms of employment status, the highest adjusted prevalences for poor-rated general health and depressive symptoms are found in the group of mothers without employment. This applies to both single mothers and mothers living in a partnership. In the group of non-employed mothers, the adjusted prevalences for both health outcomes are higher for single mothers than for mothers living in partner households. In the group of mothers with full-time employment, the adjusted prevalences for mothers of both family forms do not differ significantly. The adjusted prevalences for part-time working mothers do not differ within the family forms from the adjusted prevalences for full-time working mothers. If, on the other hand, only mothers in part-time employment are considered, the adjusted prevalences are higher for single mothers than for mothers in partner households.

With regard to social support, there are no differences in the adjusted prevalences by family type either within the group of mothers with low social support or within the group of mothers with strong social support. The adjusted prevalences for both family types with low social support are higher than those with strong social support. In the group with medium social support, however, there are greater differences in the adjusted prevalences by family type. Single mothers with medium social support are more likely to have poor general health or depressive symptoms.

**Annex Figure 1:**
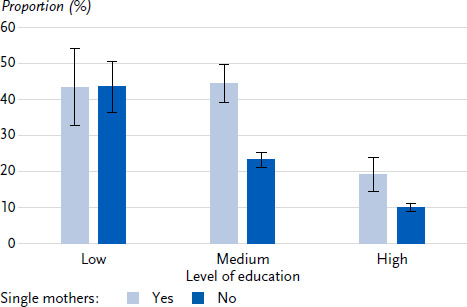
Smoking of single mothers compared to mothers living in partner households, stratified by level of education (Poisson regressions with interaction on partner status, predicted probabilities in %, 95 % confidence intervals), adjusted for age, region of residence, country of birth, year of survey, number of children and age of youngest child. Source: GEDA 2019/2020, GEDA 2021, GEDA 2022/2023 (pooled)

Regarding smoking, there is no variance in the adjusted prevalences among single parents according to income, employment status or social support. The adjusted prevalences are around 40 % in all subgroups. For mothers living in partner households, however, the adjusted prevalences vary in different ways with income, employment status and social support. When stratified according to the mothers’ level of education (Annex Figure 1), however, there are also clear differences in the adjusted prevalences for smoking among single mothers. Among single mothers, the adjusted prevalences are more than twice as high in the groups with low and medium level of education compared to the high educational group.

There are hardly any differences between income groups in terms of self-reported utilisation of professional help for mental health problems. Only in the medium-income group, the utilisation of professional help is higher among single mothers than among mothers from partner households. Professional help due to mental health problems is mainly utilised by single mothers who are not employed or receive little social support; the rate of utilisation among them is around 50 %.

## 4. Discussion

### 4.1 Summary of the results and comparison with the current state of research

Our analysis shows significantly higher prevalences for both single mothers and single fathers compared to parents in couple households in terms of self-rated general health, chronic diseases, depressive symptoms, tobacco consumption and the self-assessed need for and utilisation of professional help due to mental health problems.

Overall, the more frequent health impairments of single parents described here between 2019 and 2023 largely coincide with national and international research results at different points in time.

Several studies have shown that single mothers are more likely to rate their general health as not good compared to mothers in partner households [14, 23, 26, 44]. Sperlich et al. [23] found an increase in the differences between single mothers and mothers living in couple households in terms of poor general health between 1994 and 2018. Comparing the results of the surveys GEDA 2009 – 2012 [14] and GEDA 2019 – 2023 also shows a trend towards increasing differences in prevalence between single parents and partnered parents [14]. The difference between the prevalences of the two family forms is 8.5 % for mothers in 2009 to 2012 and 12.2 % in 2019 to 2023. For fathers, there is a difference in prevalence of 5.9 % in 2009 to 2012 and 17.8 % in 2019 to 2023. However, whether the increase in the differences is statistically significant was not examined in this article.

With regard to the observed higher prevalence of depressive symptoms among single mothers and fathers, other studies have also found greater impairment of mental health [13, 14, 45, 46]. However, the studies use different indicators and instruments to measure mental health, meaning that the prevalences are not directly comparable. The same applies to the results of the GEDA studies 2009 – 2012, which are based on respondents’ reports of depression diagnosed by a doctor or psychotherapist [14]. However, in both GEDA 2009 – 2012 and GEDA 2019 – 2023, the prevalence of depression diagnosed by a doctor in the last twelve months or depressive symptoms in the last two weeks is twice as high among single mothers and fathers compared to parents in partner households.

Studies on smoking behaviour also confirm the higher prevalence observed among single mothers [14, 28, 29] and fathers [14].

The findings of higher utilisation of professional help for mental health problems by single mothers and fathers are consistent with similar results, such as a higher use of psychotherapy by single mothers [32] and of counseling or other support services for families [31].

Secondly, the present analysis investigated whether the differences in prevalence can be attributed to single parenthood or to differences in the composition of both parent groups in terms of demographic and social determinants. For mothers, the effects of family type on self-rated health, chronic diseases, depressive symptoms and smoking are reduced, especially when controlling for income, but also for level of education and social support. Differences in employment status cannot explain the higher prevalence of single mothers for these outcomes. For fathers, on the other hand, there is a decline in the effects of single parenthood, especially when considering employment status, but also social support and, to a lesser extent, income. Level of education is not a mediator for them – except for smoking. With regard to the need for and utilisation of professional help for mental health problems, differences in social support play an important role. Overall, the more frequent health impairments among single parents can be partly, but not entirely, attributed to a more socially disadvantaged situation [13, 14, 19, 25].

However, only a small part of the possible explanatory factors could be considered in the present analysis. We did not control for various forms of strain and stress that are more common among single parents due to the often sole responsibility for the care and upbringing of the children, the double burden of family and work, and the lack of social and temporal resources [11]; this applies particularly to the period of the pandemic [10]. Previous or current stress due to conflicts with the former partner may also play a role in this context [47]. As subjectively perceived stress was not recorded in the GEDA study, it could not be included in the analysis. In studies in which subjectively perceived stress and dissatisfaction with the financial situation or available social support were considered, health differences between single parents and parents living in partner households could in part be explained entirely by these factors [13, 48, 49].

In addition to these causal effects, selection effects can also play a role. For example, it is conceivable that severe and debilitating chronic or mental illnesses in mothers and fathers can cause or increase stress and couple conflicts, which may then lead to separation and thus single parenthood [50, 51]. Long-standing partnership conflicts prior to separation may even have a greater impact on the mental and physical health of family members than the separation or divorce itself [50]. However, a longitudinal study for Germany shows that subjective health declines continuously over the duration of single parenthood [18]. This leads to the conclusion that it is not so much poor health that leads to separation, but rather single parenthood that leads to poorer health [18].

The increased need for and higher utilisation of professional help due to mental health problems can presumably be explained by higher prevalence of various mental problems, although this hypothesis was not tested in the present analysis.

Overall, it can be assumed that the various explanatory mechanisms outlined above interact with each other [18] and have different effects on the various health indicators. This requires further research with longitudinal data.

In the final step, we analysed for mothers whether the associations between partner status and health vary with income, employment status and social support. Among single mothers, there are partly large differences in the prevalence of self-rated general and mental health and in the utilisation of professional help due to mental health problems according to income, employment status and social support. The health of single mothers varies more with the social determinants mentioned than is the case for mothers living in partner households. Single parents with high incomes, full-time employment and strong social support show almost no differences in the three health outcomes compared to mothers living with a partner. This may be related, among other things, to the fact that single mothers with full-time employment and high income are more likely to have access to external services in the form of support with housework and childcare, which may help to reduce stress and improve health. In contrast, mothers at risk of poverty have the highest prevalence of poor general health and depressive symptoms. However, significant differences between family types in these two health outcomes are only evident for employment status, to the detriment of single parents. Numerous studies on multiple roles show that non-working mothers in general and single parents in particular suffer from poorer health [18, 52]. For Germany, it has also been shown that single parents benefit more in terms of self-rated health from taking up full-time employment than mothers living in partner households [36]. Although employment can lead to problems with compatibility and stress due to the limited time resources of single parents, it can also enable single parents to have greater financial independence from support services, higher self-esteem and social contacts outside the family. In the case of stress in one area of life, the other area of life may provide compensation and relief [18]. It can be assumed that these mechanisms apply more strongly to higher-educated mothers [52]. On the other hand, a selection effect can be assumed to the effect that good subjective health is a prerequisite for single parents to work full-time in addition to their family responsibilities.

Professional help for mental health problems is utilised particularly by non-employed single parents and single parents with low social support. As they have higher health burdens, the higher utilisation can be considered as ‘needs-based’.

In contrast, there are no differences in smoking among single parents in terms of income, employment status and social support. With regard to employment status and social support, this was already evident in the results of the GEDA studies 2009 – 2012. However, there was a strong social gradient with regard to socioeconomic status. The current analysis clearly shows that the differences in smoking are not associated with income, but with level of education. This corresponds to findings showing that level of education is particularly relevant for health-related behaviour [53].

In summary, the present results underline that single parents are not a homogeneous group, but that this family form is differentiated into various social and health statuses [35].

### 4.2 Limitations and strengths

The strengths of this analysis lie in the size and representativeness of the sample as well as the timeliness of the data. In addition, the data make it possible to analyse different health outcomes and social determinants and thus draw a comprehensive picture of the health status of single parents. The size of the sample allows statements to be made about the health of single fathers in a population-representative survey, although the number of cases is still too small for differentiated moderation analyses.

The main limitation of the present analysis is that the GEDA studies are cross-sectional studies that do not allow any conclusions to be drawn about the direction of the association between family type and health or health behaviour (causality vs. selection). The examination of associations is only carried out via stepwise adjustment for selected social determinants and cannot prove causality. As longitudinal data are not collected in the GEDA studies, dynamics in the family situation of single parents cannot be captured. Although this article reports results stratified by gender, it does not analyse the different reasons for single parenthood among mothers and fathers. Furthermore, the GEDA studies do not allow a differentiation of single parents with regard to the amount of childcare provided. The group referred to as single parents in this analysis also includes separated parents, without being able to explicitly identify them in the data. It was also not considered whether single parents have a partner who does not live in the same household. Therefore, the living situation of single parents cannot be captured in all its complexity in this study. For this purpose, further studies are needed that include a more differentiated coverage of separated and single parents, longitudinal data and qualitative methods.

Even though the data from the GEDA studies 2019 – 2023 includes the period of the COVID-19 pandemic, no trend analysis was carried out on changes in the health status of single parents before, during and after the COVID-19 pandemic. The numbers of single parents are too small to allow a differentiated analysis of the different phases of the pandemic.

### 4.3 Conclusions

Overall, under the societal conditions of the past five years (including the pandemic), similar results can be found regarding the health status of single parents as in previous years: Single parents have higher levels of impairment in several aspects of health. Against this backdrop, reducing the structural social disadvantage of single parents and promoting their health appears to be beneficial. Since the majority of single parents are mothers and their often precarious social situation is also the result of structural disadvantages faced by women, health promotion for single parents must also aim to reduce gender-related inequalities in health. In the Federal Framework Recommendations of the National Prevention Conference according to § 20d para. 3 SGB V, single parents are explicitly named as a target group for prevention and health promotion [54].

‘Family health promotion’ provides the conceptual framework for promoting the health of single parents [55]. The concept is based on a broad understanding of family that encompasses all intergenerational household forms, regardless of social or biological boundaries. Family health promotion aims to promote the health of family members less individually and more systemically through the family as a whole by creating health-promoting conditions for family life and strengthening family resources. With regard to single parents and their families, family health promotion measures focus on improving the framework conditions for families as well as on specific settings for single parents and strengthening social networks [55].

In the sense of the ‘Health in All Policies’ approach of the World Health Organization (WHO) [56], interventions in various policy areas (family, labour market, social, housing or financial policy) can improve the living situation of single parents and, in particular, reduce the structurally induced high risk of poverty [57]. In particular, a sustainable family policy is regarded as a central element of health promotion for single parents, which according to Bertram et al. [58] is to be understood as a triad of financial transfer, time and infrastructure policies [59].

The Berliner Landesgesundheitskonferenz (Berlin State Health Conference) lists the health goals for single parents as providing adequate childcare facilities for better reconciliation of family and work, supporting families with low incomes and combating child poverty [59]. It is important to bear in mind that single parents with younger children in particular need time for their family and may not be able or willing to work at certain times, or only to a limited extent. Bertram and Bujard therefore call for a flexibilisation of working time models over the course of life that meet the different time requirements in different stages of life and are secured by the welfare state [60]. The Verband alleinerziehender Mütter und Väter (Association of Single Mothers and Fathers) also calls for the social recognition of single parents and their children as equal family forms [61].

In addition to the social and legal framework conditions, the creation of health-promoting everyday living, learning and working conditions is another important approach [54]. Although families have a significant influence on the health of their members, they do not form an independent setting in the sense of the WHO setting approach [55]. The autonomy of families is protected by law and, therefore, families cannot be ‘processed’ with the methods of the setting approach [55]. However, families are embedded in living environments such as daycare centers, schools and communities. Implementing health promotion into these settings can relieve socially disadvantaged parents and their children and support them in leading the healthiest life possible regardless of the type of family and without stigmatising individual family forms.

At the level of promoting personal skills, there are, for example, primary preventive services (nutritional counselling, etc.), services to support socially disadvantaged families in the field of early intervention, child and youth services or family centres, as well as low-threshold counselling and mediation services for parents in critical separation processes. The Scientific Advisory Council on Family Issues at the Federal Ministry for Family Affairs, Senior Citizens, Women and Youth points out in its report ‘Parenting Together Separately’ that the increasing variety of care arrangements in separating families also increases the need for counseling [62].

In addition, the heterogeneity of the living situations of single parents should be considered through targeted services. For mothers with a high income and a higher professional position, interventions to improve the reconciliation of work and family life seem to be particularly relevant. Educationally disadvantaged or non-employed single parents may benefit more from more complex interventions that also address the issues of adequate income and further educational or occupational qualifications [cf. 35].

With regard to interventions to improve the health of single parents, a systematic review concludes that most prevention programmes to date have been predominantly behaviour-based and that structural prevention has rarely been evaluated [63]. It would be important to evaluate whether interventions for socially disadvantaged families can also improve the health status of single parents and their children and reduce their structural disadvantages [63].

## Key statement

- Single parents report health problems more often than parents in partner households.
- Income, education, employment and social support vary between family types and can explain some, but not all of the differences in health.
- The health of single mothers varies more strongly with income, employment status and social support than that of mothers in partner households.
- Poor general health and depressive symptoms are particularly prevalent among single mothers who are not employed or at risk of poverty and among those with low social support.
- Health promotion and prevention should consider the diversity of the social, economic and health situation of single parents.

## Figures and Tables

**Table 1:** Social situation of single parents and parents living in partner households (n = 7,861 women, 6,262 men). Source: GEDA 2019/2020, GEDA 2021, GEDA 2022/2023 (pooled)

	Mothers			Fathers		
	Single-parent household	Partner household	p-value	Single-parent household	Partner household	p-value
	n = 1,276	n = 6,723		n = 339	n = 6,063	
	% (95 % CI)	% (95 % CI)		% (95 % CI)	% (95 % CI)	
**Total**	**18.0** **(16.7 – 19.3)**	**82.0** **(80.7 – 83.3)**		**5.5** **(4.7 – 6.4)**	**94.5** **(93.6 – 95.3)**	
**Number of children**			< 0.001			< 0.001
1 child	59.9 (55.9 – 63.9)	44.2 (42.5 – 45.9)		67.8 (60.3 – 74.5)	46.1 (44.2 – 47.9)	
2 children	32.5 (28.8 – 36.3)	40.7 (39.1 – 42.3)		26.4 (20.5 – 33.4)	40.3 (38.5 – 42.1)	
≥ 3 children	7.6 (5.6 – 10.3)	15.2 (13.9 – 16.5)		5.8 (3.0 – 10.7)	13.6 (12.4 – 15.0)	
**Age of the youngest child**			< 0.001			< 0.001
0 – 6 years	30.2 (26.4 – 34.3)	50.1 (48.4 – 51.8)		23.6 (17.2 – 31.6)	51.0 (49.2 – 52.9)	
7 – 10 years	24.9 (21.6 – 28.6)	19.7 (18.4 – 21.0)		25.8 (19.9 – 32.9)	20.3 (18.9 – 21.8)	
11 – 17 years	44.9 (41.0 – 48.9)	30.2 (28.8 – 31.7)		50.5 (42.4 – 58.6)	28.6 (27.0 – 30.3)	
**Income**			< 0.001			< 0.001
Low	35.3 (31.3 – 39.5)	15.0 (13.6 – 16.4)		22.6 (15.8 – 31.2)	12.0 (10.7 – 13.5)	
Medium	60.5 (56.3 – 64.5)	70.4 (68.9 – 71.9)		65.0 (56.8 – 72.4)	67.8 (66.1 – 69.5)	
High	4.2 (3.1 – 5.6)	14.6 (13.7 – 15.6)		12.5 (9.2 – 16.7)	20.1 (18.9 – 21.5)	
**Level of education**			< 0.001			0.105
Low	21.1 (17.5 – 25.3)	11.9 (10.5 – 13.4)		23.5 (16.3 – 32.7)	17.5 (15.8 – 19.4)	
Medium	64.6 (60.6 – 68.4)	60.7 (59.1 – 62.3)		52.4 (44.3 – 60.4)	52.1 (50.2 – 53.9)	
High	14.3 (12.6 – 16.1)	27.4 (26.2 – 28.6)		24.0 (19.4 – 29.4)	30.4 (29.1 – 31.8)	
**Employment status**			< 0.001			< 0.001
Not employed	23.0 (19.3 – 27.1)	21.4 (19.9 – 22.9)		12.9 (7.3 – 21.6)	5.6 (4.6 – 6.7)	
Part-time	46.8 (42.8 – 50.8)	56.9 (55.2 – 58.6)		11.1 (6.5 – 18.2)	6.2 (5.4 – 7.2)	
Full-time	30.2 (26.8 – 33.8)	21.7 (20.4 – 23.1)		76.0 (67.0 – 83.2)	88.2 (86.8 – 89.5)	
**Social support**			< 0.001			0.001
Low	17.9 (14.8 – 21.6)	9.7 (8.6 – 11.0)		23.8 (16.4 – 33.3)	8.6 (7.5 – 9.9)	
Medium	45.3 (41.3 – 49.5)	41.0 (39.4 – 42.7)		43.5 (35.6 – 51.7)	44.4 (42.5 – 46.2)	
Strong	36.7 (32.9 – 40.7)	49.3 (47.6 – 51.0)		32.7 (25.8 – 40.4)	47.0 (45.1 – 48.8)	

95 % CI = 95 % confidence interval

**Table 2:** Health and health behaviour of single parents and parents living in partner households (weighted prevalences in %). Source: GEDA 2019/2020, GEDA 2021, GEDA 2022/2023 (pooled)

	Mothers				Fathers			
		Single-parent household	Partner household			Single-parent household	Partner household	
	n	Prevalence (95 % CI)	Prevalence (95 % CI)	p-value	n	Prevalence (95 % CI)	Prevalence (95 % CI)	p-value
Self-rated general health (fair to very poor)	7,998	29.7 (26.1 – 33.6)	17.5 (16.1 – 18.9)	< 0.001	6,402	32.5 (24.6 – 41.5)	14.7 (13.3 – 16.1)	< 0.001
Chronic disease	7,987	47.0 (43.0 – 51.0)	39.9 (38.2 – 41.6)	0.001	6,394	48.2 (40.1 – 56.4)	33.0 (31.2 – 34.7)	< 0.001
Depressive symptoms	7,936	19.6 (16.5 – 23.2)	9.8 (8.8 – 11 .0)	< 0.001	6,367	22.5 (15.8 – 31.0)	10.5 (9.3 – 11 .9)	< 0.001
Smoking	6,661	43.7 (39.3 – 48.3)	22.0 (20.4 – 23.7)	< 0.001	5,243	44.4 (35.6 – 53.4)	31.8 (29.8 – 33.9)	0.005
Self-assessed need for professional help	2,005	32.6 (25.8 – 40.1)	22.4 (19.7 – 25.4)	0.006	1,692	27.7 (16.6 – 42.4)	13.2 (10.7 – 16.2)	0.007
Self-reported utilisation of professional help	2,006	25.9 (19.9 – 33.0)	12.7 (10.7 – 15.1)	< 0.001	1,692	24.5 (13.2 – 40.8)	7.8 (5.9 – 10.4)	< 0.001

95 % CI = 95 % confidence interval

**Table 3:** Health and health behaviour of single mothers compared to mothers living in partner households (prevalence ratios, Poisson regressions, all models adjusted for age, region of residence, country of birth, year of survey, number of children and age of youngest child). Source: GEDA 2019/2020, GEDA 2021, GEDA 2022/2023 (pooled)

		Model 1	Model 2	Model 3	Model 4	Model 5	Model 6
	N		Model 1 + income	Model 1 + education	Model 1 + employment status	Model 1 + social support	Fully adjusted model
		PR (95 % CI)	PR (95 % CI)	PR (95 % CI)	PR (95 % CI)	PR (95 % CI)	PR (95 % CI)
Self-rated general health (fair to very poor)	7,823	**1.59** (1.36 – 1.85)	**1.38** (1.18 – 1.61)	**1.45** (1.24 – 1.69)	**1.52** (1.31 – 1.77)	**1.45** (1.23 – 1.69)	**1.27** (1.09 – 1.48)
Chronic disease	7,813	**1.15** (1.04 – 1.26)	1.10 (0.99 – 1.21)	**1.12** (1.01 – 1.23)	**1.14** (1.03 – 1.25)	**1.11** (1.01 – 1.23)	1.08 (0.98 – 1.19)
Depressive symptoms	7,767	**1.95** (1.58 – 2.40)	**1.64** (1.32 – 2.04)	**1.76** (1.42 – 2.19)	**1.85** (1.50 – 2.29)	**1.74** (1.40 – 2.16)	**1.49** (1.19 – 1.86)
Smoking	6,511	**1.81** (1.59 – 2.07)	**1.70** (1.48 – 1.95)	**1.62** (1.42 – 1.86)	**1.79** (1.57 – 2.05)	**1.77** (1.55 – 2.03)	**1.56** (1.36 – 1.80)
Self-assessed need for professional help	1,970	**1.50** (1.15 – 1.96)	**1.47** (1.12 – 1.93)	**1.53** (1.17 – 1.99)	**1.49** (1.15 – 1.94)	**1.42** (1.11 – 1.83)	**1.46** (1.13 – 1.89)
Self-reported utilisation of professional help	1,971	**1.98** (1.42 – 2.77)	**1.98** (1.40 – 2.80)	**2.00** (1.43 – 2.80)	**1.94** (1.41 – 2.66)	**1.89** (1.38 – 2.59)	**1.94** (1.40 – 2.69)

PR = Prevalence Ratio, 95 % CI = 95 % confidence interval, bold = p-value < 0.05 Reference group (PR = 1): Mothers living in a partnership

**Table 4:** Health and health behaviour of single fathers compared to fathers living in partner households (prevalence ratios, Poisson regressions, all models adjusted for age, region of residence, country of birth, year of survey, number of children and age of youngest child). Source: GEDA 2019/2020, GEDA 2021, GEDA 2022/2023 (pooled)

		Model 1	Model 2	Model 3	Model 4	Model 5	Model 6
	N		Model 1 + income	Model 1 + education	Model 1 + employment status	Model 1 + social support	Fully adjusted model
		PR (95 % CI)	PR (95 % CI)	PR (95 % CI)	PR (95 % CI)	PR (95 % CI)	PR (95 % CI)
Self-rated general health (fair to very poor)	6,235	**1.99** (1.51 – 2.62)	**1.75** (1.34 – 2.28)	**1.87** (1.45 – 2.42)	**1.70** (1.31 – 2.21)	**1.70** (1.29 – 2.23)	**1.44** (1.12 – 1.84)
Chronic disease	6,228	**1.34** (1.11 – 1.62)	**1.30** (1.08 – 1.57)	**1.33** (1.10 – 1.61)	**1.25** (1.05 – 1.49)	**1.27** (1.05 – 1.53)	1.20 (1.00 – 1.43)
Depressive symptoms	6,207	**2.31** (1.60 – 3.35)	**2.01** (1.41 – 2.86)	**2.16** (1.50 – 3.11 )	**1.95** (1.33 – 2.84)	**1.88** (1.28 – 2.77)	**1.56** (1.07 – 2.27)
Smoking	5,102	**1.52** (1.24 – 1.87)	**1.37** (1.12 – 1.69)	**1.39** (1.14 – 1.70)	**1.46** (1.19 – 1.79)	**1.46** (1.18 – 1.80)	**1.30** (1.06 – 1.59)
Self-assessed need for professional help	1,639	**2.23** (1.29 – 3.85)	**2.14** (1.25 – 3.68)	**2.22** (1.29 – 3.84)	**1.96** (1.04 – 3.68)	**2.08** (1.19 – 3.64)	**1.85** (1.00 – 3.42)
Self-reported utilisation of professional help	1,639	**2.74** (1.40 – 5.37)	**2.67** (1.36 – 5.25)	**2.74** (1.39 – 5.41)	**2.37** (1.26 – 4.46)	**2.51** (1.36 – 4.63)	**2.23** (1.18 – 4.21)

PR = Prevalence Ratio, 95 % CI = 95 % confidence interval, bold = p-value < 0.05 Reference group (PR = 1): Fathers living in a partnership

**Annex Table 1:** Overview of the GEDA studies used: versions, response rates, sample sizes in total and for the analysis. Source: GEDA 2019/2020, GEDA 2021, GEDA 2022/2023 (pooled)

Study	Wave	Version used	Response rate (RR3)^1^	n total	n analysis
GEDA 2019/2020	GEDA2019/2020 total	GEDA1920_v4	–	26,507	4,403
	GEDA2019/2020-EHIS		21.6 %		
GEDA 2021	Wave 1 – 5	GEDA21_v2	-	5,030	866
	Wave 1		17.6 %		
	Wave 2		17.6 %		
	Wave 3		19.6 %		
	Wave 4		22.5 %		
	Wave 5		17.6 %		
GEDA 2022/2023	Wave 1 – 10	GEDA22_Abt2_v1	–	33,149	5,222
	Wave 1		18.3 %		
	Wave 2		16.1 %		
	Wave 3		19.2 %		
	Wave 4		18.7 %		
	Wave 5		19.5 %		
	Wave 6		19.1 %		
	Wave 7		19.6 %		
	Wave 8		19.2 %		
	Wave 9		19.0 %		
	Wave 10		19.0 %		
	Wave 11	GEDA22_t11_v4	19.6 %	4,011	625
	Wave 12	GEDA22_t12_v3	19.8 %	3,964	616
	Wave 13	GEDA22_t13_v2	19.2 %	3,966	554
	Wave 14	GEDA22_t14_v4	18.9 %	2,008	321
	Wave 15	GEDA22_t15_v2	18.6 %	2,002	300
	Wave 16	GEDA22_t16_v2	18.7 %	2,012	287
	Wave 17	GEDA22_t17_v2	18.8 %	2,005	273
	Wave 18	GEDA22_t18_v2	18.1 %	2,007	331
	Wave 19	GEDA22_t19_v2	17.4 %	2,006	305
	Wave 19	GEDA22_t20_v2	18.9 %	2,004	327

^1^ Standards of the American Association for Public Opinion Research (AAPOR) [64]

**Annex Table 2:** Description of the sample

	Women		Men	
	n (unweighted)	% (weighted)	n (unweighted)	% (weighted)
**Total**	**7,999**	**58.9**	**6,402**	**41.1**
**Family form (partner status)**				
Single-parent household	1,276	18.0	339	5.5
Partner household	6,723	82.0	6,063	94.5
Missing	0		0	
**Age (in years)**				
15 – 29	285	7.3	147	3.6
30 – 39	2,553	43.2	1,772	35.2
40 – 49	3,722	38.9	2,789	40.5
50 – 59	1,439	10.6	1,694	20.7
Missing	0		0	
**Region of residence**				
West Germany	6,398	82.5	5,043	80.3
East Germany	1,013	13.4	937	15.5
Berlin	585	4.1	420	4.2
Missing	3		2	
**Country of birth**				
Germany	6,779	82.0	5,459	81.0
Not Germany	1,208	18.0	929	19.0
Missing	12		14	
**Year of survey**				
2019	1,140	14.7	951	14.0
2020	1,257	13.8	1,011	13.1
2021	483	6.0	416	6.2
2022	2,784	34.8	2,144	34.1
2023	2,335	30.6	1,880	32.5
Missing	0		0	
**Number of children**				
1 child	3,651	47.0	2,771	47.3
2 children	3,335	39.2	2,750	39.5
≥ 3 children	1,013	13.8	881	13.2
Missing	0		0	
**Age of the youngest child (in years)**				
0 – 6	3,097	46.5	2,944	49.5
7 – 10	1,772	20.6	1,383	20.6
11 – 17	3,130	32.9	2,075	29.8
Missing	0		0	
**Income**				
Low (< 60 %)	934	18.6	470	12.6
Medium (60 % – < 150 %)	5,457	68.6	4,207	67.7
High (≥ 150 %)	1,608	12.7	1,725	19.7
Missing	0		0	
**Level of education**				
Low	443	13.6	496	17.9
Medium	3,661	61.4	2,492	52.1
High	3,880	25.0	3,399	30.0
Missing	15		15	
**Employment status**				
Not employed	1,256	21.6	235	6.0
Part-time employed	4,524	55.1	443	6.5
Full-time employed	2,206	23.2	5,719	87.5
Missing	13		5	
**Social support**				
Low	581	11 .2	419	9.5
Medium	3,173	41.8	2,772	44.3
Strong	4,108	47.0	3,072	46.2
Missing	137		139	
**Self-rated general health**				
Fair/poor/very poor	1,320	19.7	813	15.6
Very good/good	6,678	80.3	5,589	84.4
Missing	1		0	
**Chronic disease**				
Yes	3,256	41.2	2,110	33.8
No	4,731	58.8	4,284	66.2
Missing	12		8	
**Depressive symptoms**				
Yes	746	11 .6	5,858	11.2
No	7,190	88.4	509	88.8
Missing	63		35	
**Smoking**				
Yes	1,263	25.9	1,226	32.5
No	5,398	74.1	4,017	67.5
Missing	1,338		1,159	
**Self-assessed need for professional help**				
Yes	503	24.2	232	14.0
No	1,502	75.8	1,460	86.0
Missing	5,994		4,710	
**Self-reported utilisation of professional help**				
Yes	322	15.1	136	8.7
No	1,684	84.9	1,556	91.3
Missing	5,993		4,710	
